# From egg to “no-body”: an overview and revision of developmental pathways in the ancient arthropod lineage Pycnogonida

**DOI:** 10.1186/s12983-017-0192-2

**Published:** 2017-02-07

**Authors:** Georg Brenneis, Ekaterina V. Bogomolova, Claudia P. Arango, Franz Krapp

**Affiliations:** 1 Wellesley College, Neuroscience Program, 106 Central Street, Wellesley, MA 02481 USA; 2 Moscow State University, Moscow, 119991 Russia; 3 Queensland Museum, Biodiversity Program, PO Box 3300, South Brisbane, QLD 4101 Australia; 4 Zoologisches Forschungsmuseum A. Koenig, Adenauerallee 160, D-53113 Bonn, Germany

**Keywords:** Sea spider, Evolution, Arthropoda, Embryology, Gastrulation, Postembryonic development, Anamorphic development, Evo-devo, Protonymphon larva

## Abstract

**Background:**

Arthropod diversity is unparalleled in the animal kingdom. The study of ontogeny is pivotal to understand which developmental processes underlie the incredible morphological disparity of arthropods and thus to eventually unravel evolutionary transformations leading to their success. Work on laboratory model organisms has yielded in-depth data on numerous developmental mechanisms in arthropods. Yet, although the range of studied taxa has increased noticeably since the advent of comparative evolutionary developmental biology (evo-devo), several smaller groups remain understudied. This includes the bizarre Pycnogonida (sea spiders) or “no-bodies”, a taxon occupying a crucial phylogenetic position for the interpretation of arthropod development and evolution.

**Results:**

Pycnogonid development is variable at familial and generic levels and sometimes even congeneric species exhibit different developmental modes. Here, we summarize the available data since the late 19^th^ century. We clarify and resolve terminological issues persisting in the pycnogonid literature and distinguish five developmental pathways, based on (1) type of the hatching stage, (2) developmental-morphological features during postembryonic development and (3) selected life history characteristics. Based on phylogenetic analyses and the fossil record, we discuss plausible plesiomorphic features of pycnogonid development that allow comparison to other arthropods. These features include (1) a holoblastic, irregular cleavage with equal-sized blastomeres, (2) initiation of gastrulation by a single bottle-shaped cell, (3) the lack of a morphologically distinct germ band during embryogenesis, (4) a parasitic free-living protonymphon larva as hatching stage and (5) a hemianamorphic development during the postlarval and juvenile phases. Further, we propose evolutionary developmental trajectories within crown-group Pycnogonida.

**Conclusions:**

A resurgence of studies on pycnogonid postembryonic development has provided various new insights in the last decades. However, the scarcity of modern-day embryonic data – including the virtual lack of gene expression and functional studies – needs to be addressed in future investigations to strengthen comparisons to other arthropods and arthropod outgroups in the framework of evo-devo. Our review may serve as a basis for an informed choice of target species for such studies, which will not only shed light on chelicerate development and evolution but furthermore hold the potential to contribute important insights into the anamorphic development of the arthropod ancestor.

## Background

Arthropod evolution has led to an overwhelming species richness, which goes hand in hand with an extraordinary disparity of morphological forms (e.g., [1]). When attempting to unravel the evolutionary transformations that underlay the appearance of this multitude of arthropod forms, the study of development can contribute significant insights (e.g., [2]).

Given the extreme arthropod diversity, it is not surprising that development of many taxa has not been investigated in nearly as much detail as in groups with long-standing laboratory model organisms. Pycnogonida, also known as Pantopoda or sea spiders, is one of these understudied taxa. Although they have since their first description fascinated and puzzled their students – including the Nobel prize-winning founder of *Drosophila* genetics T.H. Morgan [3] – investigations of sea spider development remain to this day relatively scarce.

**Fig. 1:**
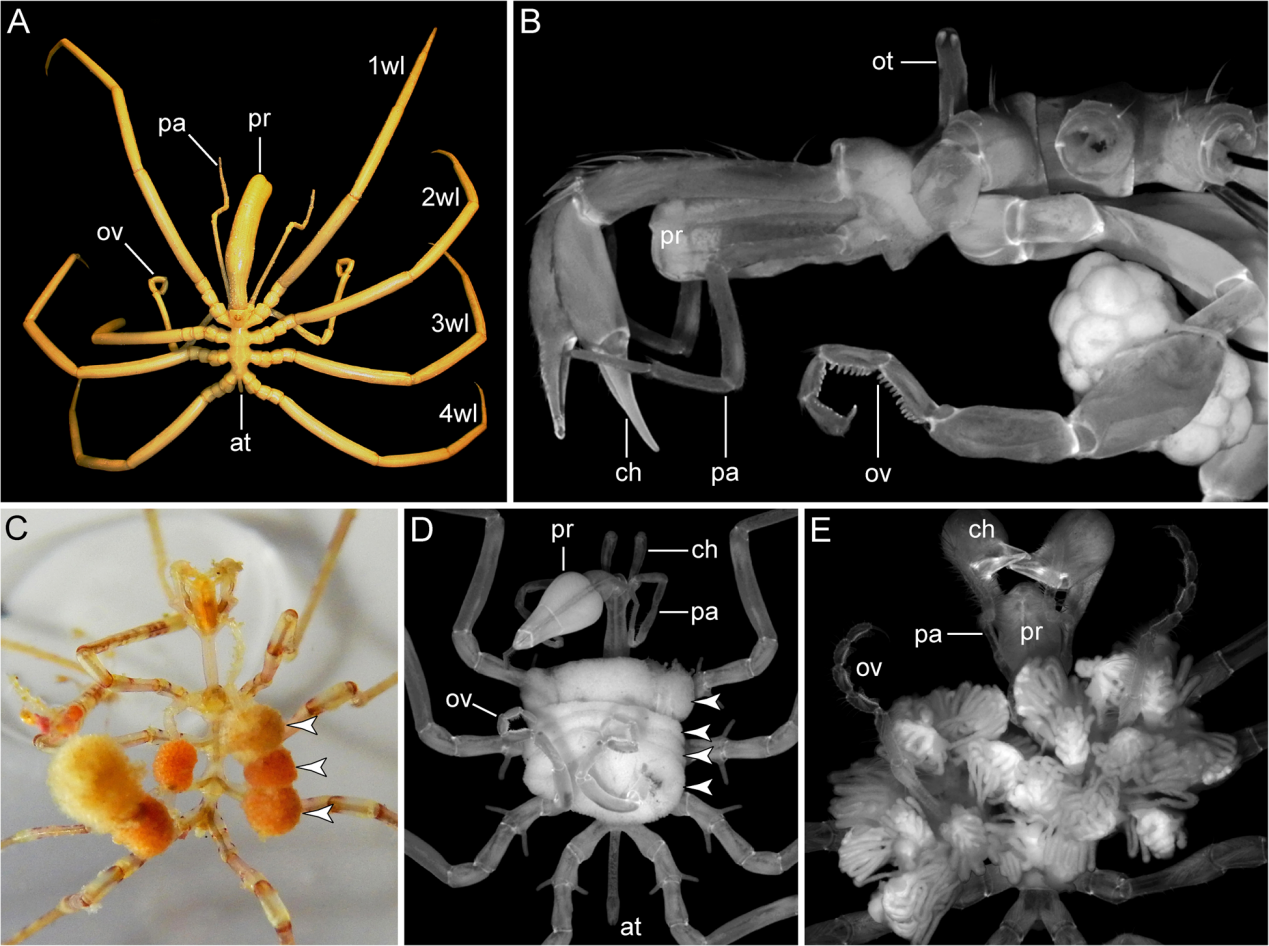
Adult morphology of Pycnogonida and male paternal brood care. **a** *Colossendeis australis*, dorsal view. Note small body and prominent proboscis and long walking legs. **b** *Nymphon australe*, lateral view of anterior body region of an egg-carrying male, autofluorescence image. For better view of proboscis, cheliphores, palps and ovigers, the walking legs have been removed. **c** *Nymphon molleri*, ventral view of live male carrying egg packages (*arrowheads*) of different matings on each oviger. Note color change of egg packages from proximal (*orange*) to distal (*light yellow*) along the oviger, being indicative of different developmental stages of the embryos. **d** *Ascorhynchus ramipes*, ventral view of male carrying four egg packages (*arrowheads*). Note that both ovigers insert into each of the midline-spanning packages. **e** *Nymphon micronesicum*, ventral view of male carrying far advanced postlarval instars, autofluorescence image. In some pycnogonid species, the offspring leaves the male’s ovigers only at far advanced developmental stages

Due to their rather peculiar adult morphology, which features an unusually small and often tube-like body that contrasts starkly to a prominent anterior proboscis and four pairs of long spindly walking legs (Fig. 1a), pycnogonids are occasionally nicknamed the “no-bodies”. However, contrary to the insignificance suggested by this sobriquet, sea spiders are one of the pivotal taxa to take into consideration when reconstructing the evolutionary transformations along the first bifurcations of the arthropod tree of life. Extant pycnogonids are nowadays widely accepted as the descendants of one of the oldest arthropod lineages, which diverged from their next closest surviving relatives in the Cambrian (ca. 500 million years ago, e.g., [4]). Although their exact phylogenetic position is still not entirely beyond debate (see [5] for a history of the discussion), recent analyses recover sea spiders within the Chelicerata, as sister group to all remaining extant chelicerate taxa (e.g., [6–8]; see [1] for review). Accordingly, a better understanding of pycnogonid development has been recognized to hold “great potential to inform on chelicerate evolution and development more generally” [9].

The last three decades have seen comparably few new investigations on aspects of embryonic development in sea spiders [10–13], which have nonetheless added important new insights to the histological studies from the late 19^th^ and the 20^th^ century [14–18]. By contrast, significantly more studies have investigated postembryonic development (e.g., [19–22]). Differences between the postembryonic development of some pycnogonid lineages were recognized long ago (e.g., [16, 23, 24]) and some more recent works have compiled data and distinguished several developmental pathways (e.g., [19, 25, 26]), with Bain [25] giving a good overview of the literature on postembryonic development up to the time of publication. However, there are persisting terminological inconsistencies and the need for clarity in the definition of each developmental pathway that has been proposed in earlier summaries and more recently based on new data.

Here, we first summarize key features of sea spider reproduction and embryonic development briefly, before focusing on the postembryonic period. We present a synthesis of previous ideas and propose a more consistent terminology with clearer definitions. The redefined developmental pathways are based on (1) the type and anatomy of the hatching stage, (2) developmental-morphological characteristics during subsequent postembryonic development and (3) selected life history features. Based on these key features and on the current hypotheses on internal phylogenetic relationships, we discuss possible evolutionary developmental trajectories within Pycnogonida.

### A primer to pycnogonid biology

With less than 1500 described species, Pycnogonida is a comparably small group by arthropod standards. However, many recent morphological and molecular studies illustrate that the taxonomy of traditional pycnogonid families, genera and even species needs to be critically approached and that actual diversity is hitherto underestimated, with new species being described on a regular basis (e.g., [27–34]). In this review, species names have been updated according to [35].

Sea spiders are restricted to marine habitats, in which they mostly inhabit the epibenthos, and are encountered at all latitudes and in all depths, including even deep sea hydrothermal vents (e.g., [36]). Their presence is often not apparent at first glance, since many species are of small size and cryptic in the benthic communities, where they prey on sessile or slow-moving and predominantly soft-bodied invertebrates, often cnidarians but also bryozoans, mollusks, echinoderms or polychaetes [37, 38]. The life cycle of many (but not all) pycnogonids includes different host/prey species during different phases (early postembryonic instars vs. juveniles/adults). This, coupled to the small size of early postembryonic stages and a comparably slow development, renders the establishment of successfully reproducing laboratory cultures challenging and time-consuming. To this day, there are only very few species for which the complete life cycle has been investigated in the laboratory (e.g., *Pycnogonum litorale* [10, 11, 39–41]; *Propallene longiceps* [42–44]; *Nymphon hirtipes* [45]).

### Adult morphology of Pycnogonida

Without exception, adult pycnogonids are equipped with an anterior proboscis (Fig. 1) and typically also with an anterodorsal ocular tubercle bearing two pairs of eyes (Fig. 1b). The proboscis is flanked by the first limb pair, the generally three-articled and raptorial cheliphores (Fig. 1b and e), being followed by the sensory palps and the ovigers, both limb pairs displaying various article numbers in different taxa (Fig. 1b, d and e). The ovigers are used by the males to carry developing eggs (Fig. 1b–d) and sometimes also hatched postembryonic instars (Fig. 1e) – a rare example of paternal brood care in invertebrates – but in some taxa also for grooming and/or other functions (see [46]). Notably, not all pycnogonids retain the complete set of these three anterior limb pairs in the adult: with the exception of the ovigers in males, all of them can be partially or completely reduced in a taxon- and sex-specific pattern (e.g., Fig. 1a and d). Posterior to the ovigers, the walking legs are borne on lateral processes of the body segments (Fig. 1a). While most species have four pairs of walking legs, instances of five or six pairs occur in some taxa (e.g., [37, 47]). The legs show a remarkably conserved composition across extant pycnogonid taxa, being comprised of nine articles, which are (from proximal to distal) coxae 1, 2, and 3, femur, tibiae 1 and 2, tarsus, propodus and terminal claw (or main claw). Due to the limited space in the pycnogonid body, long diverticula of the midgut and the gonads are displaced far into the legs and most (but not all) pycnogonids have segmentally repeated gonopores, which are always located on coxa 2. Posteriorly, the last walking leg segment features an unsegmented anal tubercle (Fig. 1a and d) that bears distally the anus and is generally interpreted as the vestige of a formerly multisegmented posterior body region (e.g., [9, 48]). The latter notion is also supported by fossils that have been placed in the pycnogonid lineage (e.g., [49–51]).

### Egg size and egg number

During mating, fertilized eggs are transferred from the female to the ovigers of the male, where they are glued into packages with secretions of cement glands located in the male's femora (see [46] for review). The egg packages are carried on the ovigers at least until hatching of the first postembryonic instar (Figs. 1b-e and 3a). For some taxa, a polygamous mating system has been documented (e.g., *Achelia simplissima* [52]) and males may bear several egg packages stemming from different matings, either separately on each oviger (e.g., Ammotheidae, Endeidae, Nymphonidae, Callipallenidae; Fig. 1c) or with both ovigers together (e.g., some Ascorhynchidae; Fig. 1d). In other groups, only one massive package from a single mating is carried by the male at a time (e.g., Pycnogonidae). While some species are known to reproduce repeatedly over the course of several years (e.g., *Pycnogonum litorale* [40]), others have been indicated to die after one reproductive season (e.g., *Nymphon hirtipes* [45]).

Significant differences in the yolk amount per egg and correspondingly in egg sizes are encountered among and within taxa (e.g., [53]; see Table 1). As a general rule, egg size is negatively correlated to the egg number produced by the female. In the case of small eggs with low yolk content, more than 1000 eggs may be given off during a single mating (r-strategy; e.g., Phoxichilidiidae, Endeidae, Pycnogonidae), whereas big yolky eggs are produced in significantly lower numbers (K-strategy; Callipallenidae, some species of Nymphonidae, Ammotheidae, Pallenopsidae). As already noted by Meinert [24], egg size can be taken as an indicator of the duration of lecithotrophic nutrition in postembryonic life. In species with large eggs, at least the first postembryonic instars rely on their yolk reserves and switch to active feeding only later in development. In representatives with small eggs, active feeding as parasites of soft-bodied invertebrates starts soon after hatching.

**Table 1:** Range of egg sizes of species belonging to various pycnogonid taxa

Taxon	Species	Egg diameter [μm]	Source
Ammotheidae	*Achelia echinata*	75	[17]
	*Tanystylum orbiculare*	80	[14]
	*Tanystylum intermedium*	60	[53]
	*Nymphonella tapetis*	70	[79]
	*Ammothella tuberculata*	67.5	[89]
Pallenopsidae	*Pallenopsis hodgsoni*	>600	Brenneis pers. observation
Nymphonidae	*Nymphon spinosum*	600	[53]
	*Nymphon brevicaudatum*	600	[93]
	*Nymphon gracilipes (“N. fuscum”)*	120–150	[93]
	*Nymphon macrum (“N. brevicollum”)*	260	[93]
Callipallenidae	*Callipallene brevirostris*	250–280	[14, 60]
	*Callipallene emaciata*	~200	[17]
	*Propallene longiceps*	300	[42]
	*Propallene kempi*	400–500	[109] (most likely erroneous)
		~200	Brenneis pers. observation.
	*Parapallene avida*	~250	[92]
	*Neopallene* sp.	450	[53]
	*Meridionale* sp.	~300	Brenneis pers. observation.
Endeidae	*Endeis spinosa*	50–60	[17, 60]
Phoxichilidiidae	*Anoplodactylus angulatus*	30	[17]
	*Anoplodactylus erectus*	30	[89]
	*Anoplodactylus jonesi (“A. antillianus”)*	27–36	[37]
	*Anoplodactylus eroticus*	~40	[86]
	*Phoxichilidium femoratum* *(“P. maxillare”, “P. tubulariae”)*	~50	[14, 87]
Pycnogonidae	*Pycnogonum litorale*	~130	[10]

### Embryonic development of Pycnogonida

Regardless of egg size, embryonic development of pycnogonids is characterized by a holoblastic cleavage [10, 14–17, 42, 54].

**Fig. 2:**
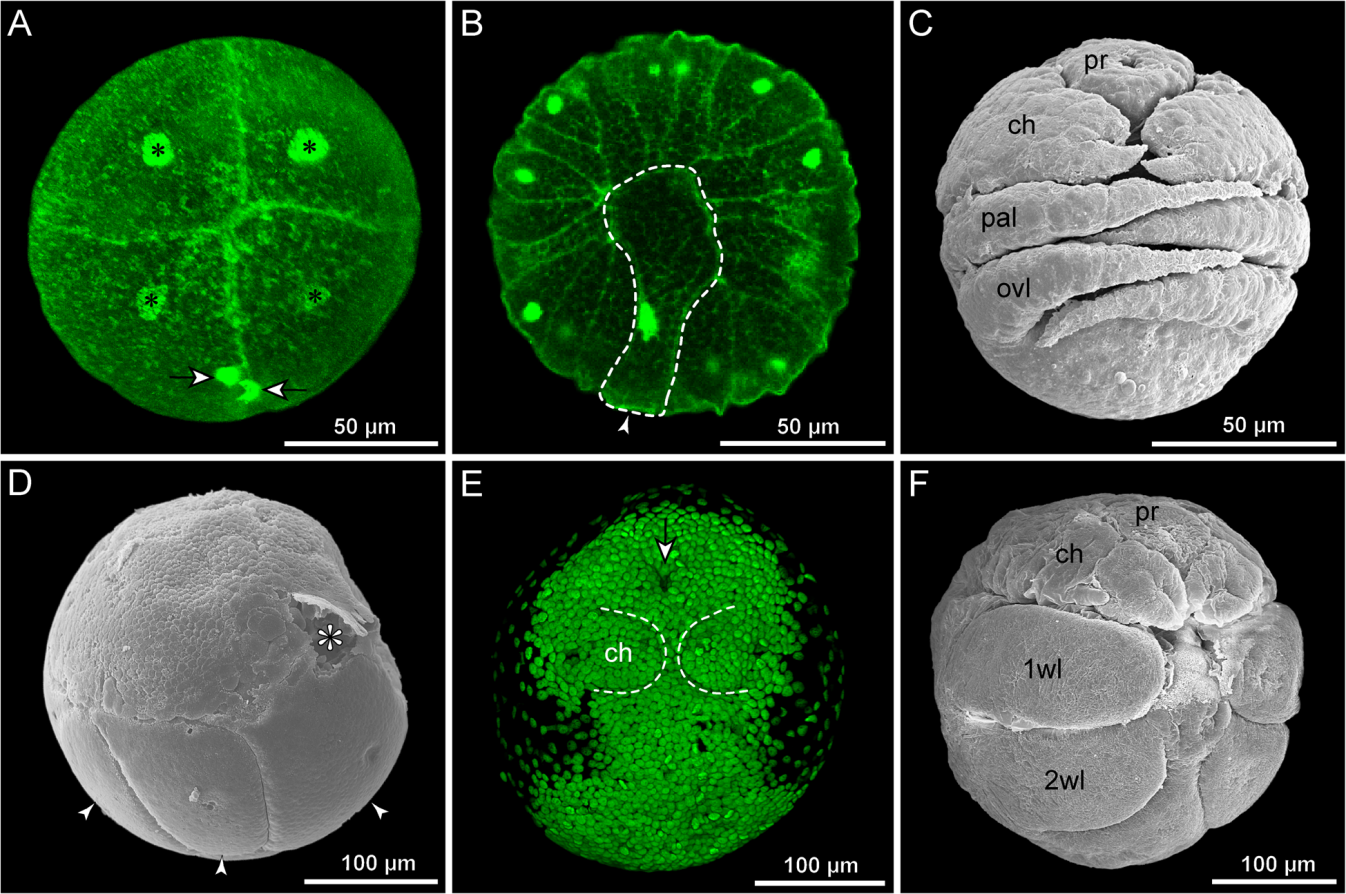
Embryonic development of Pycnogonida. **a**-**c** *Pycnogonum litorale* (Pycnogonidae), representing ‘small egg’ pycnogonids. **a** Four cell stage (Sytox nucleic acid staining). The blastomeres are of equal size. Asterisks mark cell nuclei, *arrows* indicate two brightly stained granules. **b** Initiation of gastrulation (Sytox nucleic acid staining). Note the immigration of the large bottle-shaped cell that is still attached to the embryo’s surface (*arrowhead*). **c** Embryonic morphogenesis (SEM). In the shown developmental stage, the proboscis, cheliphores and palpal and ovigeral larval limbs of the prospective protonymphon larva are recognizable. **a**&**b** modified from [10] and reproduced with permission of Springer; **c** modified from [11] and reproduced with permission of John Wiley and Sons. **d-f** *Meridionale* sp. (Callipallenidae), representing ‘large egg’ pycnogonids. **d** Early germ band stage (SEM). One embryonic hemisphere is covered by the densely packed small germ band cells, whereas the other hemisphere features few large yolk-rich cells (*arrowheads*). Asterisk indicates a damaged region. **e** Slightly later germ band stage (Sytox nucleic acid staining). Note stomodeum (*arrow*) in a far anterior position, being posteriorly followed by the cheliphore limb buds. Scattered nuclei around the germ band illustrate successive overgrowing of the large yolk-rich cells of the other embryonic hemisphere. **f** Late embryonic morphogenesis (SEM). Note that *Meridionale* sp. hatches as an advanced postlarva and develops walking leg pairs 1 and 2 before hatching. **d**&**f** modified from [12] and reproduced with permission of Springer

In species with small to medium-sized eggs (diameter < 200 μm, Fig. 2a–c), early cleavages result in equal-sized blastomeres (Fig. 2a), which are arranged in an irregular pattern. A recent study on *Pycnogonum litorale* highlighted considerable variations in spindle orientations and asynchronous blastomere divisions, which is strongly indicative of an indeterminate cleavage [10, 55]. Gastrulation is initiated by the immigration of a single bottle-shaped cell (Fig. 2b) [10, 16, 54] followed by immigration and epiboly of a number of smaller cells. It has yet to be traced in detail, which of these cells (and their progeny) give rise to which prospective entodermal and mesodermal structures [10]. Subsequent embryonic development does not feature a “proper” germ band at any stage and embryonic morphogenesis (e.g., [11]) and organogenesis (e.g., [15]) lead to the formation and hatching of a protonymphon larva (Fig. 2c; see below).

By contrast, representatives of some taxa (Callipallenidae, some Nymphonidae, Ammotheidae, Pallenopsidae) have large yolk-rich eggs (diameter ≥ 200 μm, Fig. 2d–f) and unequal cell divisions are observed early on, starting sometimes even with the very first cleavage (e.g., [14, 16, 17]). The resulting blastomere asymmetry could be indicative of an early cell determination, but blastomere arrangements in later stages have not been reported to show a stereotypic pattern. However, rigorous cell lineage studies are pending. The early blastomere asymmetry translates subsequently into an arrangement of small densely packed cells in the prospective ventral embryonic hemisphere (germ disc) and the persistence of slowly dividing, large yolk-rich cells in the other hemisphere (Fig. 2d) [12, 17]. Classical histological studies have characterized the gastrulation as epiboly (e.g., [16]), detailed observations obtained with modern techniques are lacking. The germ disc develops into a germ band (Fig. 2e, “intermediate germ” according to [55]), the margins of which continue to extend and overgrow the yolk-rich cells during subsequent embryonic morphogenesis [12].

Reinvestigations of stomodeum and proboscis formation during embryonic morphogenesis of “small egg species” as well as “large egg species” show that the stomodeum is formed distinctly anterodorsal to the cheliphoral limb buds [10–12]. Only subsequent morphogenetic movements result in the pre-/paroral position of the first limb pair in relation to the outgrowing proboscis. In support of one of the earliest descriptions [23], proboscis formation does not seem to involve a structure that can be homologized with the labrum (upper lip) of other arthropods [11, 12]. This renders pycnogonids the only arthropod taxon without an identifiable labrum.

With regard to embryonic organogenesis, progress has been made at the level of nervous system development. The cellular processes underlying neurogenesis have been shown to exhibit similarities to different arthropod groups [13]. Among others, the involvement of a neural stem cell type – as indicated in previous histological studies (e.g., [14, 18]) – could be confirmed in advanced stages of neurogenesis. This finding might question the validity of neural stem cells as an apomorphy of hexapods and (some) crustaceans [13, 56]. Importantly, however, gene expression, gene function and cell lineage studies are needed to gain deeper insights not only into these neural stem cells but also into all other aspects of pycnogonid development. As of now, such investigations are almost completely missing (but see [57, 58]).

### The protonymphon larva – the most common pycnogonid hatching stage

Postembryonic development of pycnogonids is always indirect, encompassing a series of instars (the term used here to denote developmental stages separated by intermittent molts). More specifically, the great majority of studied pycnogonids show a hemianamorphic postembryonic development (as defined in [59]), which features an anamorphic phase (=with segment addition per molt) followed by an epimorphic phase (=no further segment addition per molt). The actual molting process has been observed only in a few laboratory cultures (e.g., [39, 44], but see [22]) and the occurrence of molts is usually inferred from morphological differences between instars.

**Fig. 3:**
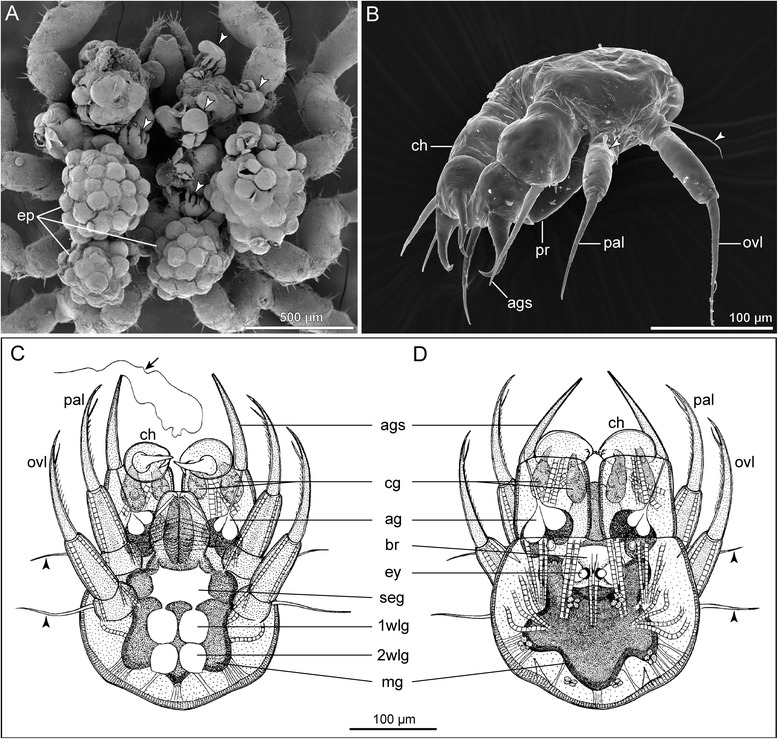
The protonymphon larva of Pycnogonida. **a** Ventral view of egg-carrying male of *Tanystylum* sp., SEM (modified from [73], therein published as *“Tanystylum bealensis”,* reproduced with permission of John Wiley and Sons). *Arrowheads* mark newly hatched protonymphon larvae. **b** Anterolateral view of protonymphon larva of *Achelia assimilis*, SEM (modified from [63], reproduced with permission of Cambridge University Press). *Arrowheads* mark gland processes of the palpal and ovigeral larval limbs. **c**, **d** Internal anatomy of the protonymphon larva of *Nymphon brevirostre* (modified from [61], reproduced with permission of Springer). *Arrowheads* mark gland processes of palpal and ovigeral larval limbs. The *arrow* highlights thread-like secretion of the cheliphoral attachment gland. **c** Ventral view. **d** Dorsal view

In most taxa, the hatching stage is a protonymphon larva (Fig. 3), first named so by Hoek [60]. This larva has an externally unsegmented body that bears a dorsomedian pair of pigmented eyes, a larval proboscis and just three limb pairs: the larval cheliphores and two additional larval limbs (Fig. 3b–d; e.g., [61–63]). According to neuroanatomical data [15, 64] these limb pairs are affiliated with the deutocerebrum and the two following segmental neuromeres of the larval nervous system. Together with larval *Hox* gene expression patterns [57, 58] this supports the homology of the pycnogonid cheliphore and the chelicera of other chelicerates. Since the larval limb pairs following the cheliphores correspond in position and segmental innervation (even if not in structure and function) to the adult palps and ovigers, they are here referred to as palpal and ovigeral larval limbs.

The larval cheliphore is comprised of three articles: the proximal scape and the two more distal ones, which form a chela (Fig. 3b). The palpal and ovigeral larval limbs are uniramous and three-articled as well, their distalmost article being generally claw-shaped (Fig. 3b–d; exception: Phoxichilidiidae, see below).

Posterior to the ovigeral larval limbs, the hind body is fairly undifferentiated. Internally, it comprises the anlage of the first walking leg segment (in some species even that of the second walking leg segment), as evidenced by the presence of primordia of the segmental ventral ganglia (Fig. 3c; e.g., *Achelia borealis* [65], *Nymphon brevirostre* [61]). Externally, however, it shows no signs of segmentation and only in some species, a slight elevation of the walking leg 1 primordium may be discernible at the posterior body pole. Dorsal to the developing ventral nerve cord, the midgut represents a blind ending sac – hindgut and anus are not yet developed (Fig. 3c and d). Anteriorly and posteriorly directed midgut extensions may indicate the anlagen of the midgut diverticula of cheliphores and future walking legs 1 (Fig. 3d).

Typically, an attachment gland is located in the cheliphore’s scape (Fig. 3c and d), being connected to a hollow spine on the scape. Thread-like secretions are released through this spine, by means of which the larva either secures attachment to its invertebrate host or remains fixed on the father’s oviger. Correspondingly, the palpal and ovigeral larval limbs may each bear a flexible spine with a pore on the proximal article (Fig. 3b-d; e.g., *Ammothella biunguiculata* [66]), being connected to a gland suggested to be serially homologous to the cheliphoral attachment gland [15, 67]. However, the function of these palpal and ovigeral glands is unknown.

In addition, the chela itself often houses another set of glands (Fig. 3c and d) that open to the outside via a pore on each of the chela fingers [15, 19, 61, 67]. An involvement of the chela glands in feeding or defense has been suggested but not yet conclusively proven [16, 68].

### The larval, postlarval and juvenile phases of pycnogonid development

Postembryonic development after hatching can be subdivided into three different phases: the larval, postlarval and juvenile phase.

#### The larval phase

**Fig. 4:**
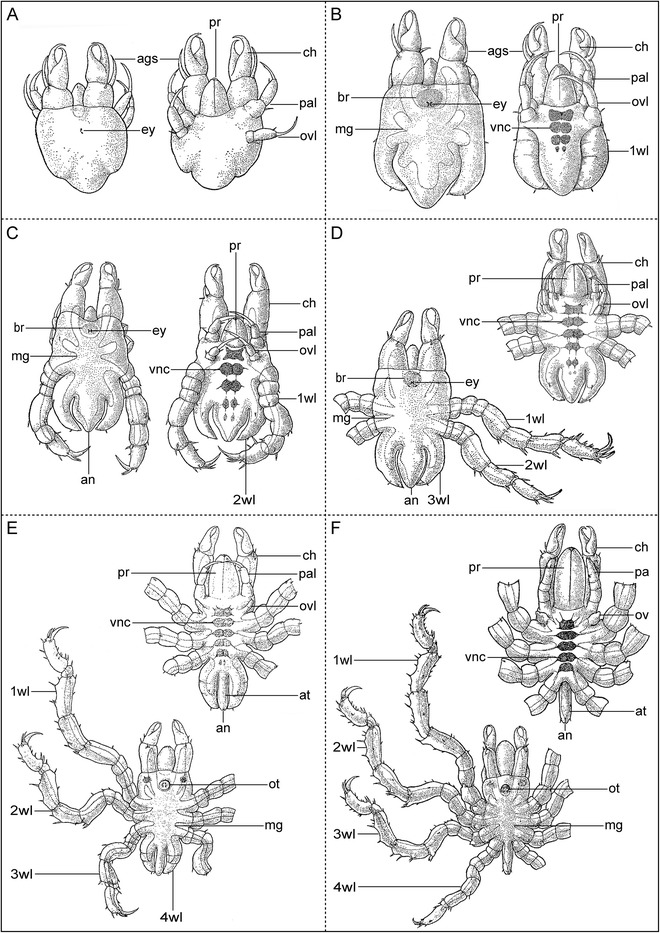
Type 1 postembryonic development of Pycnogonida. **a**-**f** Sequence of postembryonic instars of *Achelia alaskensis* up to the first juvenile instar (modified from [70], reproduced with permission of Hokkaido University). Dorsal view always on the left side, ventral view on the right side. Note strictly sequential development of the walking legs. The late protonymphon larva in (**a**) shows slight elevations of walking leg pair 1 posterior to the ovigeral larval limb (potentially the second larval instar of postembryonic development, the actual hatching having not been observed)

This phase includes those instars that closely resemble the protonymphon larva as described above (Fig. 4a). Species-dependently, it encompasses only the hatching first instar or additionally also the second one (*Tanystylum orbiculare* [14]; *Nymphon gracile* [17]; *Pycnogonum litorale* [41]; *Achelia gracilipes* [69]).

#### The postlarval phase

In the majority of species, the postlarval phase encompasses the anamorphic molts of the postembryonic development and is always characterized by the formation and further differentiation of the walking leg segments with their substructures. Characteristic larval features are still retained during (parts of) this phase. For instance, the cheliphoral attachment gland and its associated spine often remain functional in the first postlarval instars. Likewise, the structure of three-articled palpal and ovigeral larval limbs may at first stay unchanged, but soon after the anterior walking leg pairs become functional they decrease in size and gradually atrophy (especially the ovigeral larval limbs) (Fig. 4b–e). The timing of walking leg segment development varies between different pycnogonid groups (see below). Most commonly, each walking leg differentiates via three external stages, separated by two intermittent molts. An unarticulated elongate limb bud is followed by an intermediate seven-articled leg (with “femur-tibia 1” and “tarsus-propodus” precursor articles), which then finally transforms into the nine-articled adult leg (e.g., *Tanystylum orbiculare* [14]; *Nymphon unguiculatum* [20]). Slight deviations from this pattern are documented in some species (see [22] for an overview).

As in the protonymphon larva, the formation of the ventral segmental ganglia continues to predate limb bud outgrowth in each walking leg segment (e.g., [65, 70]). Thus, the complete number of segmental ganglia is already discernible in instars with an incomplete set of walking leg anlagen (Fig. 4c and d). Addition of new neural cells to the growing ganglia continues during the entire postlarval and also in the subsequent juvenile phase (potentially even still in adults). The regions of neural cell production (“neurogenic niches”) correspond to the “ventral organs” described in classical histological studies [14, 16, 71]. Extant pycnogonids develop one or two additional small ganglia in late postlarval instars, which then fuse with the last walking leg ganglion (Fig. 4d and e; e.g., [71]).

Soon after walking limb bud outgrowth, the corresponding midgut diverticulum begins to extend into it (Fig. 4). Data on the timing of hindgut and anus formation are scarce. To all appearances, these events are related to the beginning of active feeding, which varies significantly between postembryonic developmental pathways (see below).

Reliable information on the location of the primordial germ cell(s) in the larval stages is missing, but the paired gonad anlagen become recognizable in the early postlarval phase in a dorsal position at the border of walking leg segments 1 and 2 [72]. From that point on, they continue to differentiate and expand through the trunk and into the walking legs [14, 16, 72].

#### The juvenile phase

The transition from postlarval to juvenile phase is here based on the molt that leads to a “miniature adult” with the full number of functional walking legs (although the last pair might still lack the complete article number) (Fig. 4f). In most known cases, this represents the first epimorphic molt.

In the juvenile phase, the cheliphoral attachment gland and its spine are lacking. The palpal and ovigeral larval limb pairs start to transform into the adult structures, i.e., they grow gradually out into the palps and ovigers (if present in the adult) or are completely atrophied (e.g., Fig. 4f). Also the proboscis and cheliphores attain their definite adult structure, which leads in some taxa to a partial (e.g., Tanystylidae [73]) or even complete cheliphore reduction (e.g., Colossendeidae: Fig. 1a; Pycnogonidae [41]). The ocular tubercle has become more prominent and bears by now the final number of eyes (sometimes already during late postlarval phase) (Fig. 4e and f). The complete through-gut is formed and terminates with the functional anus at the distal tip of the anal tubercle, which is found in its definite orientation. Due to ongoing gonad expansion and maturation, distinguishing advanced juvenile instars (sometimes called subadults) from mature adults can be challenging. In this phase, external changes after molts may be minimal and mainly limited to an increase of overall body size. Hence, it has been difficult to determine whether a fixed number of species- and sex-specific juvenile molts occur before sexual maturity. Speaking against this, four independent investigations of the development of *Pycnogonum litorale* [16, 39–41] indicate that the number of juvenile molts varies, ranging from normally five to seven (for both sexes), to exceptionally eight or even nine. Additionally, low temperature and starvation have been shown to increase the duration of intermolt intervals [40].

Apart from visible mature oocytes in the gonads of females or the bearing of egg packages by males, the most important morphological indicator of sexual maturity is the presence of gonopores on the second coxae.

### From hatching to adult: five pathways of postembryonic development

**Fig. 5:**
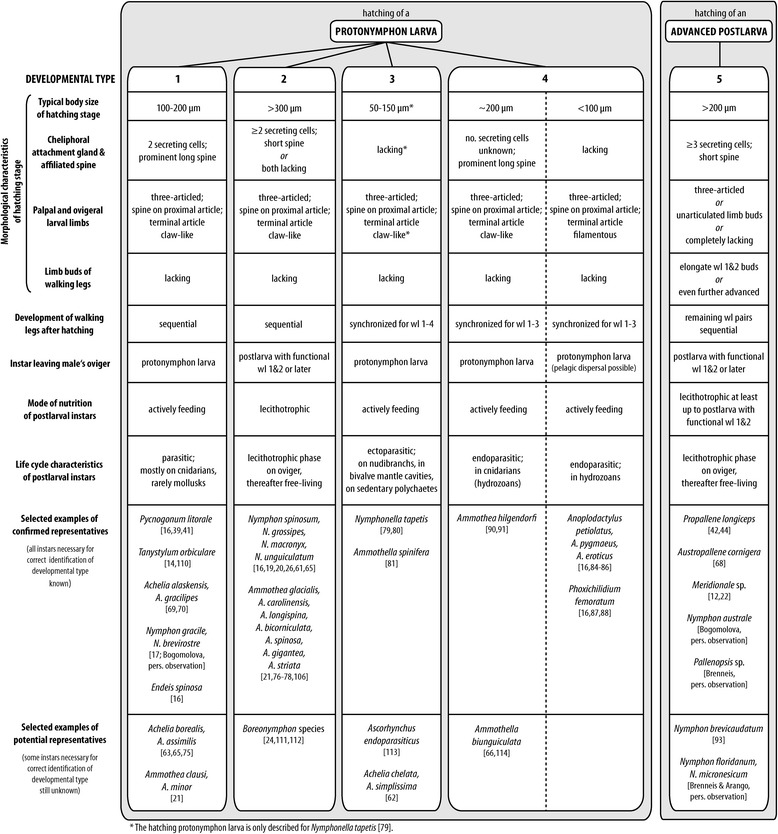
Overview of the different modes of postembryonic development in Pycnogonida. The general structure of the diagram is adopted from [19] but was extended and modified to accommodate additional details and terminological changes [110–114]

Figure 5 provides an overview of several key characteristics of the five different pathways of postembryonic development in pycnogonids. While types 1 to 4 share a protonymphon larva as the hatching stage, type 5 is characterized by the hatching of an advanced postlarva.

#### Type 1: Parasitic development with sequential differentiation of walking legs

(Figs. 4 and 5)

This type corresponds to type 1 of Dogiel [74] and Sanchez [17], the “typical protonymphon” pathway of Bain [25] and the “ectoparasitic” mode of Burris [62].

The eggs and hatching protonymphon larvae are generally of medium size (roughly 100–200 μm) but exceptions are found, e.g., in Endeidae (*Endeis spinosa* [17, 60] with an egg diameter of 50–60 μm). The scape of the larval cheliphore bears an elongate attachment gland spine that may project beyond the chela tips. The attachment gland comprises exactly two large secreting cells, which also act as reservoirs for the secretion product. Frequently, the hatching larva abandons the father’s ovigers, but offspring may also stay attached to the oviger for one or two molts and leave as postlarval instars with limb buds of the first walking leg pair (e.g., *Achelia borealis* [65, 75]). The postlarval instars feed actively as parasites. The great majority of investigated species are ectoparasitic, but some cases of apparent endoparasitic development have been reported (e.g., *Achelia alaskensis* [70]). The walking leg segments are formed sequentially during the anamorphic molts along a pronounced anterior-posterior developmental gradient, whereby each leg pair differentiates according to the mentioned three-stage-sequence (see above).

In laboratory cultures of *Pycnogonum litorale* – the best investigated representative of developmental type 1 – five molts from protonymphon larva to the last postlarval instar have been observed [39, 41]. Development up to this fifth molt took on average 83 days at 15 °C water temperature [39]. Adults of this species were observed to live for up to 9 years in laboratory cultures [40].

#### Type 2: Lecithotrophic development with sequential differentiation of walking legs

(Figs. 5 and 6)

**Fig. 6:**
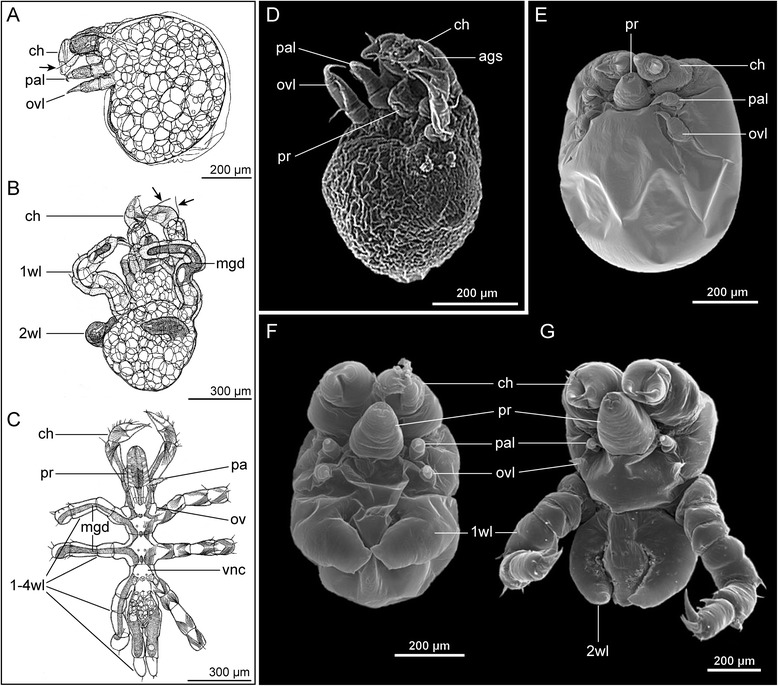
Type 2 postembryonic development of Pycnogonida. **a**-**c** Three attaching postembryonic instars of *Nymphon grossipes* (modified from [65]). Arrows mark thread-like secretions of the cheliphoral attachment gland. **a** Lecithotrophic protonymphon larva, lateral view. **b** Postlarval instar with articulated walking leg 1 and limb bud of walking leg 2, ventral view. **c** Oldest attaching instar, a late postlarva with articulated walking legs 1–3 and elongate limb bud of walking leg 4 (the latter considered as three-articled in [65]), ventral view. **d** Lecithotrophic protonymphon larva of *Nymphon unguiculatum*, ventral view, SEM. **e** Lecithotrophic protonymphon larva of *Ammothea carolinensis*, ventral view, SEM. **f**, **g** Postlarval instars 1 and 2 of *Ammothea bicorniculata*, ventral views, SEM. Note increasing reduction of palpal and especially ovigeral larval limbs. **d**-**g** modified from [20, 21] and reproduced with permission of Springer

This type corresponds to type 3 of Dogiel [74], is included in the “attaching larva” pathway of Bain [25] and represents the “lecithotrophic protonymphon” mode of Bogomolova and Malakhov [26] and the “prolonged attaching” mode of Burris [62].

This developmental mode has been observed only in some representatives of Nymphonidae and Ammotheidae. The eggs and hatching protonymphon larvae are large and exceed 300 μm in all reported cases. The protonymphon larva is equipped with a copious amount of yolk that is contained in the sac-like midgut anlage (e.g., [16, 26]). Hence, the posterior body region is significantly more massive as compared to a larva of developmental type 1. Also the first or even all following postlarval instars are lecithotrophic and remain attached to the father’s ovigers. In nymphonids, attachment to the oviger is secured by the thread-like secretions of the cheliphoral attachment glands, which comprise two or more secreting cells and release the secretions at the tip of an inconspicuous short spine [16, 19, 20, 26]. Furthermore, the larval limbs are actively used to cling to the oviger and egg package remnants. Ammotheid larval and postlarval instars belonging to developmental type 2 lack the cheliphoral attachment gland spine (and presumably also the gland), active grasping being their only means to secure attachment to the male [21, 76–78]. The formation of the walking leg segments is strictly anamorphic and the legs themselves develop in a three-stage-sequence. In nymphonids, the offspring leaves the oviger frequently as late as the last postlarval instar, whereas in ammotheids, the oldest documented stage attached to the oviger is a postlarval instar with only two functional walking leg pairs.

Recently, the first successful laboratory culture of a deep sea representative has been established for *Nymphon hirtipes* [45]. In this species, embryonic development alone lasts for about 4 months and subsequent postembryonic development up to the last postlarval instar (which is leaving the father’s oviger) takes five additional months. Based on available studies, five to six molts from protonymphon larva to the first juvenile instar can be estimated (e.g., [19, 20]).

#### Type 3: Ectoparasitic development with synchronous differentiation of walking legs

(Figs. 5 and 7)

**Fig. 7:**
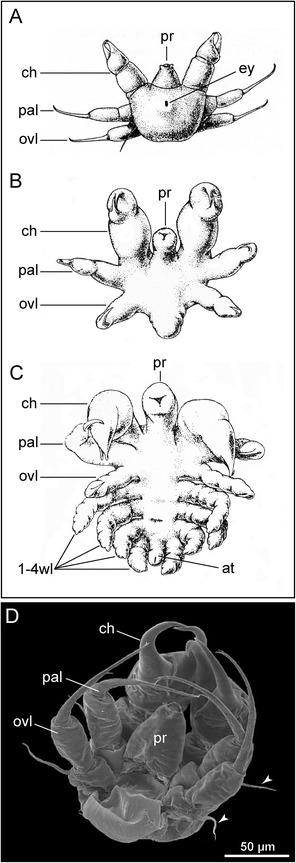
Type 3 postembryonic development of Pycnogonida. **a**-**c** Three postembryonic instars of *Nymphonella tapetis*, parasitizing in the mantle cavity of the lamellibranch bivalve *Paphia philippinarum *(modified from [79]). **a** Newly hatched protonymphon larva 1, dorsal view. **b** Presumable postembryonic instar 2 (modified protonymphon larva 2), ventral view. **c** Older postlarval instar, ventral view. Note incompletely differentiated walking leg pairs 1–4. **d** Protonymphon larva of *Achelia chelata*, its further developmental having been suggested to follow type 3*,* ventral view, SEM (modified from [62] and reproduced with permission of Cambridge University Press). *Arrowheads* mark gland processes of palpal and ovigeral larval limbs

This type corresponds to the “atypical protonymphon” pathway of Bain [25] and the incorrectly labeled “endoparasitic” mode of Burris [62].

In comparison to the other postembryonic pathways, this type of development remains poorly documented and, as of now, has been encountered only in Ammotheidae. The newly hatched protonymphon larva has been observed in a single species (*Nymphonella tapetis* [79]). It hatches from small eggs of 70 μm diameter. The three-articled cheliphore lacks an attachment gland spine and probably also the attachment gland itself. The few reported representatives have been found to parasitize in the mantle cavity of bivalves [79, 80], on sedentary polychaetes living in tubes [81], or on nudibranchs [82]. Contra Burris [62], this developmental mode should be still considered ectoparasitic instead of endoparasitic, since none of the postembryonic instars penetrate into the interior of the host body. The first parasitizing instar bears considerable resemblance to a protonymphon larva, but appears to have lost the external articulation of the limbs, although terminal claws may be still present. In contrast to developmental types 1 and 2, the walking leg segments develop almost synchronously, with only a very slight advance in the more anterior limbs. Accordingly, some molts of the postlarval phase are epimorphic. Also the stepwise differentiation sequence of the legs seems to be missing. Notably, in *Nymphonella tapetis*, neither the palpal nor the ovigeral larval limbs are atrophied. Rather, the adult palps and ovigers arise directly via gradual elongation and articulation of the larval limbs of the first parasitizing instar.

No published report on a successful laboratory culture is available. The number of molts during postembryonic development is undocumented but the described stages of *Ammothella spinifera* point to at least six [81]. In *Nymphonella tapetis*, the number might be lower (see [79]).

#### Type 4: Endoparasitic development with partially synchronous differentiation of walking legs

(Figs. 5 and 8)

**Fig. 8:**
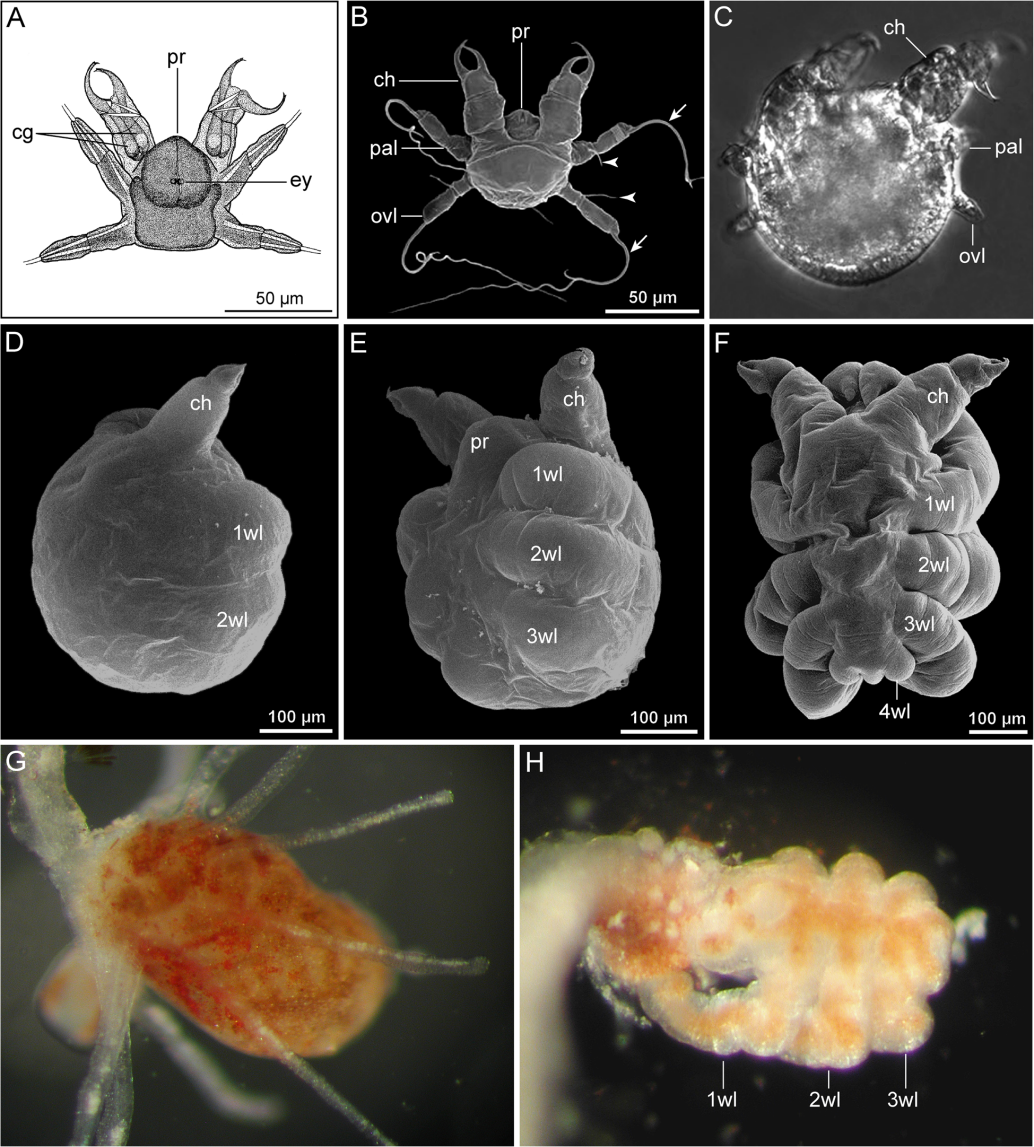
Type 4 postembryonic development of Pycnogonida. **a** Newly hatched protonymphon larva 1 of *Phoxichilidium femoratum*, ventral view (modified from [65]). **b**-**f** Sequence of larval and postlarval instars of *Anoplodactylus eroticus,* endoparasitic in the hydrozoan *Pennaria disticha*. SEM (**b**, **d**-**f**) and brightfield (**c**) micrographs (modified from [86]). Reproduced with permission of Amy Maxmen. **b** Newly hatched protonymphon larva 1, dorsal view, note modified filamentous terminal articles of palpal and ovigeral larval limbs (*arrows*). *Arrowheads* mark gland processes of palpal and ovigeral larval limbs. **c** Postembryonic instar 2 (=modified protonymphon larva 2), dorsal view. Note significant reduction of palpal and ovigeral larval limbs. **c** Instar with primordia of walking leg pairs 1 and 2, lateral view. **d** Slightly later than (**c**), ventrolateral view. Note distinct limb buds of walking leg pairs 1–3 and lack of walking leg 4 primordia. **e** Old postlarval instar, shortly before molt and emergence from the hydranth, dorsal view. Note elongate anlagen of walking leg pairs 1–3 and tiny limb bud of walking leg 4. **g** Hydranth of live *Pennaria disticha*, infested by *A. eroticus* (Original: Amy Maxmen). **g** *A. eroticus* old postlarval instar (compare to (**f**)) protruding from ruptured hydrant of *P. disticha*. (Original: Amy Maxmen). Note orange color of the midgut diverticula extending into the walking legs

This type corresponds to type 2 of Dogiel [74] and Sanchez [17], the “encysted larva” pathway of Bain [25], and the “encysting” mode of Burris [62].

All Phoxichilidiidae belong to this developmental type. They possess the smallest reported eggs and a characteristic protonymphon larva (<100 μm in body size). The larval proboscis is very prominent and the larval cheliphores lack the attachment gland and its spine. The terminal articles of the palpal and ovigeral larval limbs are elongated and filamentous, which may facilitate locomotion (“walking”) over benthic communities and/or floating and dispersal in the pelagic zone, as suggested by larvae of *Phoxichilidium femoratum* found in plankton samples [83]. They are also used to hold on to the host [38]. Predominantly, hydrozoan polyps are infested, but parasitism of hydromedusae has also been described [84, 85]. The larva molts upon encountering a suitable host, which is then entered by the second instar [86]. In some phoxichilidiids, endoparasitic instars are encysted in the host tissue, but in others they are encountered freely in the gastrovascular cavity (e.g., [38, 86, 87]). Hence, we discourage the use of the terms “encysted” [25] or “encysting” [62] to designate this pathway as a whole (see also [19]). The first endoparasitic stage (= second instar) is characterized by significantly reduced, unarticulated palpal and ovigeral larval limbs, but can still be considered a larval stage due to the undifferentiated posterior body region. During the postlarval phase, the limb buds of walking leg pairs 1–3 arise along a very weak anterior-posterior developmental gradient (e.g., *Anoplodactylus eroticus* [86]), but their further elongation and differentiation is synchronized, whereas the anlagen of walking leg pair 4 lag distinctly behind. The last postlarval instar emerges through the body wall of the host (e.g., [16, 87–89]).

Reports of a laboratory culture of a phoxichilidiid species are lacking. In *P. femoratum*, only four molts are described for the complete development from protonymphon larva to the emerging juvenile, this period lasting in total less than 21 days [87].

Notably, a single ammotheid has been conclusively shown to follow a similar endoparasitic pathway (*Ammothea hilgendorfi* [90]). Interestingly, the protonymphon larva of this species lacks the distinctive features of its phoxichilidiid counterpart and represents basically a larva of developmental type 1 [91].

#### Type 5: Postembryonic development with hatching of an advanced postlarva

(Figs. 5 and 9)

**Fig. 9:**
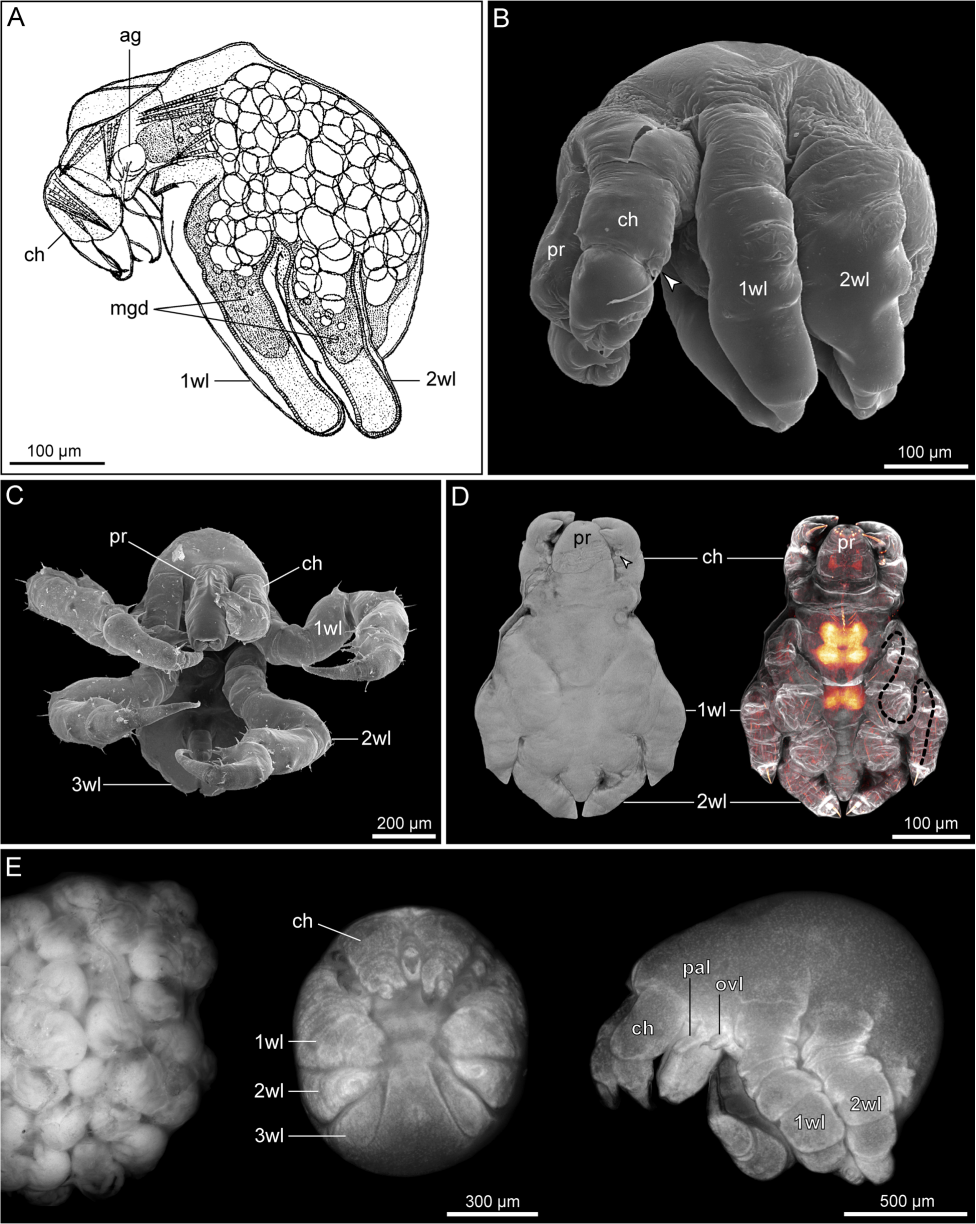
Type 5 postembryonic development of Pycnogonida. **a** Newly hatched postlarva of *Pseudopallene spinipes*, lateral view (modified from [65]). **b**, **c** Postlarval instars of *Meridionale* sp., SEM (modified from [22], reproduced with permission of Springer). **b** Newly hatched postlarva, lateral view. *Arrow head* marks short cheliphoral attachment gland spine with protruding thread-like secretions. **c** Postlarval instar 2, ventral view. This instar leaves the oviger and commences active feeding. **d** Hatching postlarva of *Propallene kempi*, ventral view. Left: surface of the postlarval cuticle through which anlagen of walking leg pairs 1 and 2 can be discerned. Right: combination of autofluorescence (*white*) and fluorescent marker FM1-43FX (*glow*). Walking leg pairs 1 and 2 underlie the cuticle, being extremely compressed and curved (*black dashed line* for walking leg 1). *Glowing* regions represent ventral nerve cord ganglia. **e** *Pallenopsis hodgsoni*. Left: autofluorescence image of egg package containing postlarvae about to hatch. Center: ventral view of late embryo (propidium iodide staining) showing anlagen of three walking leg pairs. Right: lateral view of hatched postlarva (propidium iodide staining). Note the presence of palpal and ovigeral larval limbs and the elongate walking leg pairs 1 and 2 still folded at the ventral side

This type corresponds to type 3 of Dogiel [74] and Sanchez [17], the “attaching larva” pathway of Bain [25] and the “attaching” mode of Burris [62].

Hatching stages with advanced development of walking leg segments occur in all investigated Callipallenidae (e.g., [17, 44, 68, 92]) and in some nymphonids (e.g., [93]; Bogomolova, personal observation) and pallenopsids (Brenneis, personal observation). They hatch from large yolk-rich eggs (diameter ≥ 200 μm, in nymphonids and pallenopsids > 500 μm) and are lecithotrophic with a voluminous yolk-filled midgut anlage. Previously, these stages have been termed “attaching larvae” (e.g., [25, 44, 62, 68]) since they remain attached to the father’s oviger after hatching. However, this behavior is not exclusive to them (see types 1 and especially 2) and hence this name is discouraged. Likewise, the term “walking leg-bearing larva” [22] is here discouraged, and we adopt the more general name “advanced postlarva”, which acknowledges that the developmental level of the hatching stages corresponds to postlarval instars of other pycnogonids. Obviously, all pycnogonids hatching as advanced postlarva lack the larval phase in their development.

Simultaneously to hatching, the postlarva sheds an embryonic cuticle (e.g., [12, 17, 44]). It features at least the limb buds of walking legs 1 and 2 [44, 63, 68] but in some species, elongate unarticulated walking legs 1–3 plus a small limb bud of walking leg 4 are already present [14, 22, 23]. This latter case, as found, for instance, in all investigated species of the genus *Callipallene*, represents thus an exception to the hemianamorphic theme – all postembryonic molts are epimorphic. The hatching postlarva lacks fully pigmented eyes and an open anus and remains attached to the father’s oviger for at least one additional molt. Attachment is achieved via strong threads of the cheliphoral attachment gland that comprises three or more secreting cells [24, 26, 65]. Hatching postlarvae of pallenopsids possess small but fully developed palpal and ovigeral larval limbs (Fig. 9e), but nymphonid representatives feature only a limb bud at the position of the palpal larval limb, and callipallenids lack distinct buds of larval limbs completely. If posterior body segments are still missing at hatching, they form sequentially and their walking legs follow the typical three-stage-development [22, 44, 68]. The earliest stage known to abandon the father is a postlarval instar with two functional walking leg pairs (*Propallene longiceps* [44]), but in other species it may be only the last postlarval or even a juvenile instar that leaves the oviger (e.g., *Callipallene brevirostris*, *C. emaciata* [14, 18]).

In a laboratory culture of *P. longiceps*, five molts (including shedding of embryonic cuticle) were observed from hatching to the first juvenile instar. Up to the mature adult, a total of nine molts occur, the entire development from fertilized egg to adult lasting about 5 months [44].

### The evolution of the different developmental pathways in Pycnogonida

#### Fossils, phylogenies and the ancestral mode of pycnogonid development

From a comparative developmental perspective, the five postembryonic pathways share notable correspondences, representing variations of a common hemianamorphic theme, in which mainly the relative timing of events relating to the forming walking leg segments is modified. Type 5 with its more pronounced embryonization of development deviates most from the others due to the complete lack of the protonymphon larva, but shares nonetheless many similarities with regard to the developmental sequence of segmental substructures (e.g., early development of segmental ganglia, pattern of walking leg segmentation). This leaves still the open question, which of the five pathways has retained most plesiomorphic features of the development of the pycnogonid stem species.

**Fig. 10:**
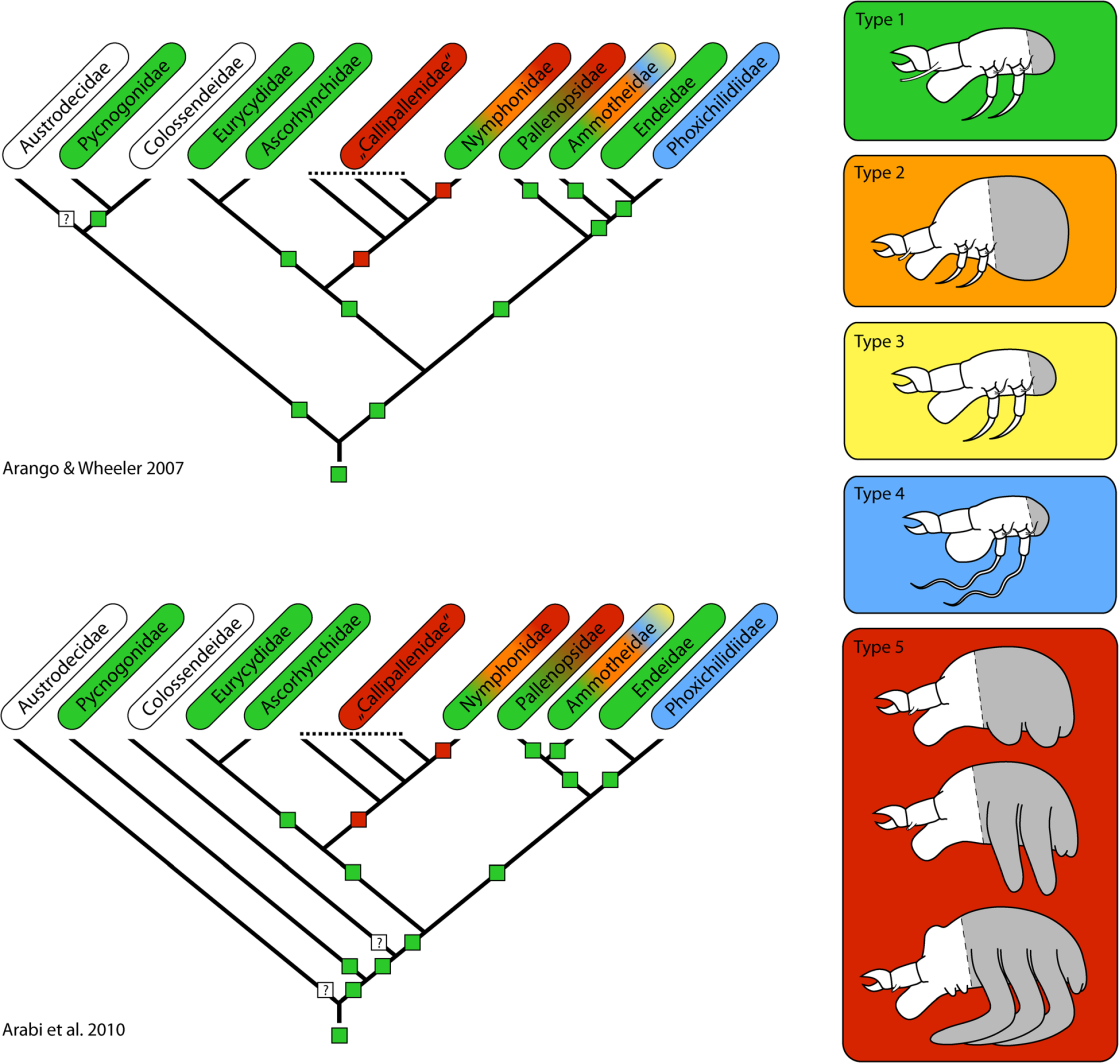
Distribution and evolution of different developmental pathways in Pycnogonida. The shown cladograms have been simplified from [96] and [97]. On the right, the different developmental types are indicated by schematic drawings of their hatching stages and a color code. The gray area in each drawing indicates the post-ovigeral body region from which the walking leg segments develop. The developmental pathways have been mapped on the cladograms according to their color code. Note that in the case of Ascorhynchidae and Eurycydidae developmental type 1 has been inferred based on hatching protonymphon larva only, since no descriptions of subsequent postembryonic development exist. Taxa names with white background indicate that no developmental data are available. In both shown scenarios, developmental type 1 (*green*) has been given preference during the reconstruction of the single nodes whenever it is found in one of the two sister groups in question (therefore also the reconstruction of type 1 as an ancestral feature in scenario two). Accordingly, only the controversial grouping of paraphyletic callipallenids with respect to nymphonids results in developmental type 5 as their ancestral developmental pathway

The most widespread developmental pathway in extant pycnogonids is type 1, being encountered across many taxa (Fig. 10). Based on this, key features of this type have been suggested as being plesiomorphic for the pycnogonid crown-group as a whole, including (1) small to medium-sized eggs, (2) a holoblastic, irregular cleavage with equal-sized blastomeres in the earliest cleavage rounds, (3) gastrulation that is initiated by the immigration of a single bottle-shaped cell, (4) the lack of a morphologically distinct germ band during embryogenesis, (5) the hatching of a parasitic and free-living protonymphon larva with a cheliphoral attachment gland and corresponding elongate spine, and (6) a hemianamorphic development during the postlarval and juvenile phases [10, 12, 55, 59, 94].

In general agreement with this notion, the oldest allegedly pycnogonid fossil – the Cambrian *Cambropycnogon klausmuelleri* [95] – has been described as a postlarval instar with three anterior limb pairs (presumably homologous to cheliphores plus palpal and ovigeral larval limbs) and just a single pair of elongate limb buds (presumably anlagen of first walking legs). Also the body length (~270 μm) corresponds well to a comparable postlarval instar of extant representatives of developmental type 1 (e.g., *Pycnogonum litorale*: 260 μm [41]). Thus, the discovery of *Cambropycnogon* seems to support an anamorphic postembryonic development as an ancient pycnogonid feature. However, it has to be cautioned that all described fossil specimens belong to a single instar only, and neither an earlier protonymphon-like larva, nor further advanced postlarval or juvenile instars are known. Accordingly, direct fossil evidence for a protonymphon-like larva without walking leg anlagen – dating back to the Cambrian or any later geological age – is still missing.

Yet, not only the fossil record but also extant sea spiders leave us with some persisting gaps of knowledge: For the three pycnogonid taxa Austrodecidae, Colossendeidae and Rhynchothoracidae neither mating behavior, nor embryonic or early postembryonic development have ever been documented. This is especially astounding in the case of the large-sized colossendeids – which are cosmopolitan, relatively diverse and frequently collected – and leads to the suspicion that this group may exhibit a deviating mode of reproduction and development that completely lacks paternal brood care [25, 37, 93]. Coincidentally, the two hitherto most comprehensive phylogenetic analyses [96, 97] indicate that Austrodecidae and Colossendeidae might have diverged relatively close to the base of the pycnogonid crown-group (if not even at the base itself, see Fig. 10). Additionally, a basal position of colossendeids within pycnogonids has also received some support from the analysis of the mitochondrial genome [98] (but only a very limited number of taxa are included). In light of this, the lack of developmental data in these taxa needs to be borne in mind when drawing conclusions regarding ancestral developmental patterns of Pycnogonida.

As of now, developmental type 1 remains uncontested as most plausible ancestral pathway of pycnogonid development (Fig. 10). However, with new data and a more reliable pycnogonid phylogeny, some of the features currently considered plesiomorphic for sea spider development may yet turn out to have evolved only *within* the pycnogonid crown-group.

#### Multiple transitions from parasitic to lecithotrophic protonymphon larvae during pycnogonid evolution

To date, developmental type 2 with a lecithotrophic protonymphon larva has been described only in some nymphonids and ammotheids, both taxa also containing species following developmental type 1. Apart from the yolk-related size increase of the protonymphon larva and the correlated prolonged lecithotrophic nutrition, type 2 is closest to type 1, with no major changes in the sequence or timing of developmental events (apart from the nutrition-related differentiation of the hindgut and anus). It seems therefore plausible that an evolutionary switch from type 1 to type 2 may have occurred independently within both pycnogonid taxa (Fig. 10). Interestingly enough, some representatives described as type 1 show the “beginning” of lecithotrophic nutrition in the first postembryonic instars (e.g., *Achelia borealis* [65, 75]); thus, type 1 and type 2 might well represent the extremes of a more continuous distribution.

Notably, developmental type 2 is documented predominantly in species living in cold waters as opposed to species of temperate or tropical latitudes. Accordingly, the switch to a more pronounced K-strategy via lecithotrophic nutrition and prolonged attachment of the offspring has been suggested to be an adaptation to low temperature habitats [25, 94, 99]. Yet, since type 1 representatives coexist in the same environments, lecithotrophic nutrition may well be a favorable but not an indispensable life history feature for pycnogonid survival in the cold.

Without a reliable internal phylogeny for nymphonids or ammotheids, independent type 1-to-type 2 transitions within each group remain a possibility. However, as of now, reports of lecithotrophic protonymphon larvae in ammotheids remain restricted to Antarctic species of the genus *Ammothea*. Further, all of these *Ammothea* species lack the characteristic cheliphoral attachment gland spine and most likely also the corresponding gland itself. This indicates a single evolutionary switch to lecithotrophic developmental type 2 in the genus *Ammothea*, coupled to an apomorphic loss of the attachment gland and spine in the larva.

#### Endoparasitic development is apomorphic for Phoxichilidiidae and a derived trait in the pycnogonid tree

The endoparasitic developmental type 4 is encountered in all phoxichilidiids. It can be unequivocally characterized by the unique – and therefore likely apomorphic – morphology of the protonymphon larva, the short duration of the development and the low number of molts. Phoxichilidiidae has been repeatedly recovered well-nested in the pycnogonid tree, as sister group to Endeidae [96, 97, 100]. Both taxa encompass pronounced r-strategists with extremely small eggs and hatching larvae. Since endeids – as unequivocally shown for *Endeis spinosa* [16, 17] – include representatives of developmental type 1, this mode seems a likely starting point for the evolution of the endoparasitic phoxichilidiid development (Fig. 10). The comparatively fast course of the latter might be an evolutionary adaptation that facilitates exploitation of hosts with distinct yearly growth periods in habitats governed by significant seasonal variations [87].

In light of the available phylogenetic studies (e.g., [96, 97]), the occurrence of a similar endoparasitic development in the ammotheid *Ammothea hilgendorfi* has to be interpreted as an independent evolutionary event. It is intriguing that this species seems to show a corresponding partially synchronized differentiation of the walking leg segments and reinvestigation of the encysting postlarval instars would be desirable, in order to assess similarities and differences to phoxichilidiids in more detail.

#### Multiple gains of embryonized development during pycnogonid evolution

Large yolk-rich eggs and the hatching of an advanced postlarva (type 5) are characteristic of all Callipallenidae, but are also found in some nymphonids (e.g., [93]; Bogomolova, personal observation) and members of the Pallenopsidae (Brenneis, personal observation).

Callipallenids and nymphonids have been recovered together in a clade [96, 97, 100, 101]. Yet, callipallenids have been recovered as a paraphyletic group due to the nested position of Nymphonidae (Fig. 10). If this controversial finding should receive further corroboration in future studies, one possible evolutionary scenario advocates the embryonization of development as a derived feature of the callipallenid-nymphonid clade, leading to a postlarva as an apomorphic hatching stage (Fig. 10). However, a reversal of this process must then have led to the re-occurrence of the protonymphon larvae of developmental types 1 and 2 within nymphonids (for discussion see [12]).

Regardless of the prevailing interpretation in the callipallenid-nymphonid case, the presence of developmental type 5 in some Antarctic *Pallenopsis* reveals at least one parallel event of embryonization of pycnogonid development (Fig. 10). The relationship of the genus *Pallenopsis* to other pycnogonid taxa has been matter of recurrent debate, having traditionally been considered a “transitional genus” between Callipallenidae and Phoxichilidiidae [47, 101–104]. In contrast to this, available phylogenetic studies generally suggest closer affinities to ammotheids and Endeidae + Phoxichilidiidae (albeit with weak support) [96, 97, 100]. Due to this and the presence of developmental type 1 in some *Pallenopsis* species, we must assume that a separate evolutionary transition from type 1 to type 5 within the genus *Pallenopsis* has taken place. A remarkable feature of pallenopsid hatching postlarvae is the presence of functional palpal and ovigeral larval limbs (Fig. 9e) as opposed to their absence or undifferentiated state in callipallenids or nymphonids, respectively. This morphological feature thus distinguishes pallenopsids from the other two pycnogonid groups with embryonized development.

### Outlook – the no-body’s contribution to arthropod evolution

The last two decades witnessed a resurgence of studies on pycnogonid postembryonic development, which provided new data and insights into the diversity of developmental types in crown-group pycnogonids. We considered it pertinent to review the data available and to resolve current inconsistencies by clarifying the terminology and delineating the different postembryonic pathways known so far. It is conceivable that new data, especially on some of the enigmatic pycnogonid groups (such as Austrodecidae and Colossendeidae) may render the re-evaluation of this scheme necessary at some point in the future. In particular the recent success of the laboratory husbandry of a deep sea nymphonid [45] holds promise for more revelations regarding the life cycle of some of the largely unstudied deep sea preferring taxa.

With no established laboratory model organism found among sea spiders, our understanding of many developmental processes at the cellular level and in terms of the underlying genetic mechanisms is still in its infancy. Clearly, additional studies are overdue and future investigations could address, among others, (1) the early embryonic development in “large egg species”, (2) the gastrulation in “large egg species” and the exact relationship of the mesodermal and entodermal cell lineages in pycnogonids in general, (3) the identification and localization of the germ line precursors during embryology, and the (4) understanding of axial growth and segmentation processes in the different developmental types. Ideally, such studies would include modern live imaging techniques, and their underpinning with gene expression and gene function data is needed. Although previous attempts to address the latter two issues have faced several challenges, first progress in the optimization of protocols has been made (e.g., [57, 86, 105]) and the by now straightforward generation of RNA seq data (or even genomes) for non-model organisms has removed several of the formerly cumbersome obstacles.

In terms of species choice for such studies, *Pycnogonum litorale* is without doubt the most promising candidate of the putatively plesiomorphic developmental type 1. Not only have successfully reproducing populations of this long-lived species been kept in the laboratory for several years (e.g., [39, 40]), but also the general course of embryonic and postembryonic development is best understood due to a series of relatively recent studies (e.g., [10, 11, 41]). By contrast, however, representatives of the further derived type 5 have the great advantage of developing part (or all) of the body segments and legs during the embryonic phase, which facilitates many investigations considerably, since embryos of different developmental stages are easily located on the males’ ovigers (as opposed to free-living postembryonic instars in type 1). Hence, a long-term laboratory culture of a type 5 species – as at least partially achieved for *Propallene longiceps* some decades ago [42–44] – would be highly desirable for pycnogonid research. Ideally, a combination of studies on both developmental types will enable the elucidation of general developmental mechanisms of crown-group sea spiders at the level of gene expression and gene function and thus pave the way for detailed comparison with available data on other arthropods and arthropod outgroups.

It is noteworthy that Pycnogonida is the only extant chelicerate taxon that shares with many crustaceans a life cycle that includes a minute marine larva with only three limb-bearing segments [e.g., [106]). The correspondence between the protonymphon larva and crustacean nauplius larva has been noted early on (see [5]) and traditionally some authors have even used it as an argument in support of a sister group relationship of both arthropod groups (e.g., [15, 16]). Even though today’s countless phylogenetic studies render this close relationship untenable (see [1] for review), it remains plausible that protonymphon and nauplius larvae have a common origin in a segment-poor larva in the life cycle of the marine arthropod ancestor [55, 107, 108]. Seen from this perspective, a renewed interest in the development of the arthropod “no-bodies” might not only shed more light on chelicerate evolution and development [9]. Beyond that – and in combination with further studies on crustaceans with nauplius larva and with new fossil evidence (e.g., [108]) – it has the potential to yield insights into the anamorphic development of the ancestor of today’s most diverse and successful animal lineage.

## Abbreviations

ag: Attachment gland
ags: Attachment gland spine
An: Anus
at: Anal tubercle
br: Brain
cg: Chela gland
ch: Cheliphore
ep: Egg package
ey: Eye
mg: Midgut
mgd: Midgut diverticula
ot: Ocular tubercle
ov: Oviger
ovl: Ovigeral larval limb
pa: Palp
pal: Palpal larval limb
pr: Proboscis
seg: Subesophageal ganglion
vnc: Ventral nerve cord
wl: Walking leg
wlg: Walking leg ganglion

## References

[1] Giribet G, Edgecombe GD, Minelli A, Boxshall G, Fusco G (2013). The Arthropoda: A Phylogenetic Framework. Arthropod Biology and Evolution Molecules, Development, Morphology.

[2] Richter S, Wirkner C (2014). A research program for Evolutionary Morphology. J Zool Syst Evol Res.

[3] Maxmen A (2008). The sea spider’s contribution to T.H. Morgan’s (1866–1945) development. J Exp Zool (Mol Dev Evol).

[4] Dunlop JA (2010). Geological history and phylogeny of Chelicerata. Arthropod Struct Dev.

[5] Dunlop JA, Arango CP (2005). Pycnogonid affinities: a review. J Zool Syst Evol Res.

[6] Regier JC, Shultz JW, Zwick A, Hussey A, Ball B, Wetzer R, Martin JW, Cunningham CW (2010). Arthropod relationships revealed by phylogenomic analysis of nuclear protein-coding sequences. Nature.

[7] Campbell LI, Rota-Stabelli O, Edgecombe GD, Marchioro T, Longhorn SJ, Telford MJ, Philippe H, Rebecchi L, Peterson KJ, Pisani D (2011). MicroRNAs and phylogenomics resolve the relationships of Tardigrada and suggest that velvet worms are the sister group to Arthropoda. Proc Natl Acad Sci.

[8] Sharma PP, Kaluziak ST, Pérez-Porro AR, González VL, Hormiga G, Wheeler WC, Giribet G (2014). Phylogenomic interrogation of Arachnida reveals systemic conflicts in phylogenetic signal. Mol Biol Evol.

[9] Schwager EE, Schönauer A, Leite DJ, Sharma PP, Wanninger A (2015). McGregor AP Chelicerata. Evolutionary Developmental Biology of Invertebrates Volume 3 Ecdysozoa I: Non-Tetraconata.

[10] Ungerer P, Scholtz G (2009). Cleavage and gastrulation in *Pycnogonum litorale* (Arthropoda, Pycnogonida): morphological support for the Ecdysozoa?. Zoomorphology.

[11] Machner J, Scholtz G (2010). A scanning electron microscopy study of the embryonic development of *Pycnogonum litorale* (Arthropoda, Pycnogonida). J Morphol.

[12] Brenneis G, Arango CP, Scholtz G (2011). Morphogenesis of *Pseudopallene* sp. (Pycnogonida, Callipallenidae) I: Embryonic development. Dev Genes Evol.

[13] Brenneis G, Stollewerk A, Scholtz G (2013). Embryonic neurogenesis in *Pseudopallene* sp. (Arthropoda, Pycnogonida) includes two subsequent phases with similarities to different arthropod groups. EvoDevo.

[14] Morgan TH (1891). A contribution to the embryology and phylogeny of the pycnogonids. Stud Biol Lab Johns Hopkins Univ Baltimore.

[15] Meisenheimer J (1902). Beiträge zur Entwicklungsgeschichte der Pantopoden. I. Die Entwicklung von *Ammothea echinata* Hodge bis zur Ausbildung der Larvenform. Z Wiss Zool.

[16] Dogiel V (1913). Embryologische Studien an Pantopoden. Z Wiss Zool.

[17] Sanchez S (1959). Le développement des Pycnogonides et leurs affinités avec les Arachnides. Archives de Zoologie Expérimentale et Générale.

[18] Winter G (1980). Beiträge zur Morphologie und Embryologie des vorderen Körperabschnitts (Cephalosoma) der Pantopoda Gerstaecker, 1863. I. Entstehung und Struktur des Zentralnervensystems. Zeitschrift für zoologische Systematik und Evolutionsforschung.

[19] Bogomolova EV (2010). *Nymphon macronyx* (Arthropoda, Pycnogonida), another pycnogonid species with “lecytotrophic protonymphon” development. Zoologiceskij Zhurnal.

[20] Cano Sánchez E, López-González PJ (2010). Postembryonic development of *Nymphon unguiculatum* Hodgson 1915 (Pycnogonida, Nymphonidae) from the South Shetland Islands (Antarctica). Polar Biol.

[21] Cano Sánchez E, López-González PJ (2013). New data concerning postembryonic development in Antarctic *Ammothea* species (Pycnogonida: Ammotheidae). Polar Biol.

[22] Brenneis G, Arango CP, Scholtz G (2011). Morphogenesis of *Pseudopallene* sp. (Pycnogonida, Callipallenidae) II: Postembryonic development. Dev Genes Evol.

[23] Dohrn A (1881). Die Pantopoden des Golfes von Neapel und der angrenzenden Meeres-Abschnitte. Fauna und Flora des Golfes von Neapel und der angrenzenden Meeres-Abschnitte.

[24] Meinert F (1899). Pycnogonida. Danish Ingolf Expedition.

[25] Bain BA (2003). Larval types and a summary of postembryonic development within the pycnogonids. Invertebr Reprod Dev.

[26] Bogomolova EV, Malakhov VV (2006). Lecithotrophic protonymphon is a special type of postembryonic development of sea spiders (Arthropoda, Pycnogonida). Dokl Biol Sci.

[27] Arango CP, Brenneis G (2013). New species of Australian *Pseudopallene* (Pycnogonida: Callipallenidae) based on live colouration, morphology and DNA. Zootaxa.

[28] Bamber RN (2013). Deep-water Pycnogonida from recent cruises to Papua New Guinea and Melanesis, with an appendix of new records form Polynesia and descriptions of five new species. Zoosystema.

[29] Dietz L, Krapp F, Hendrickx ME, Arango CP, Krabbe K, Spaak JM, Leese F (2013). Evidence from morphological and genetic data confirms that *Colossendeis tenera* Hilton, 1943 (Arthropoda: Pycnogonida), does not belong to the *Colossendeis megalonyx* Hoek, 1881 complex. Org Divers Evol.

[30] Dietz L, Pieper S, Seefeldt MA, Leese F (2015). Morphological and genetic data clarify the taxonomic status of *Colossendeis robusta* and *C. glacialis* (Pycnogonida) and reveal overlooked diversity. Arthropod Systematics Phylogeny.

[31] Staples D (2014). A revision of the callipallenid genus *Pseudopallene* Wilson, 1878 (Pycnogonida, Callipallenidae). Zootaxa.

[32] Staples D (2014). A reassessment of the pycnogonid genus *Stylopallene* (Arthropoda, Callipallenidae) with description of a new genus. Mem Mus Vic.

[33] Weis A, Meyer R, Dietz L, Dömel JS, Leese F, Melzer RR (2014). *Pallenopsis patagonica* (Hoek, 1881) - a species complex revealed by morphology and DNA barcoding, with description of a new species of *Pallenopsis* Wilson, 1881. Zool J Linnean Soc.

[34] Munilla T, Soler-Membrives A (2015). Pycnogonida from the Bellingshausen and Amundsen seas: taxonomy and biodiversity. Polar Biol.

[35] Bamber RN, El Nagar A, Arango CP. Pycnobase: World Pycnogonida Database. Available online at http://www.marinespecies.org/pycnobase. Accessed 29 Jan 2017.

[36] Arango CP, Linse K (2015). New *Sericosura* (Pycnogonida: Ammotheidae) from deep-sea hydrothermal vents in the Southern Ocean. Zootaxa.

[37] Arnaud F, Bamber RN (1987). The biology of Pycnogonida. Adv Mar Biol.

[38] Staples DA, Watson JE, Bouillon J (1987). Associations between pycnogonids and hydroids. Modern Trends in the Systematics, Ecology and Evolution of Hydroids.

[39] Behrens W (1984). Larvenentwicklung und Metamorphose von *Pycnogonum litorale* (Chelicerata, Pantopoda). Zoomorphology.

[40] Tomaschko KH, Wilhelm E, Bückmann D (1997). Growth and reproduction of *Pycnogonum litorale* (Pycnogonida) under laboratory conditions. Mar Biol.

[41] Vilpoux K, Waloszek D (2003). Larval development and morphogenesis of the sea spider *Pycnogonum litorale* (Ström, 1762) and the tagmosis of the body of Pantopoda. Arthropod Struct Dev.

[42] Sekiguchi K, Nakamura K, Onuma S (1971). Egg-carrying habit and embryonic development in a pycnogonid, *Propallene longiceps*. Zool Mag.

[43] Nakamura K, Sekiguchi K (1980). Mating behavior and oviposition in the pycnogonid *Propallene longiceps*. Mar Ecol Prog Ser.

[44] Nakamura K (1981). Post-embryonic development of a pycnogonid, *Propallene longiceps*. J Nat Hist.

[45] Mercier A, Baillon S, Hamel J-F (2015). Life history and feeding biology of the deep-sea pycnogonid *Nymphon hirtipes*. Deep Sea Res I.

[46] Bain BA, Govedich FR (2004). Mating and courtship behavior in the Pycnogonida (Chelicerata: Class Pycnogonida): a summary. Invertebr Reprod Dev.

[47] Hedgpeth JW (1947). On the evolutionary significance of the Pycnogonida. Smithsonian Miscellaneous Collections.

[48] Brenneis G, Schmidt-Rhaesa A, Harzsch S, Purschke G (2016). Pycnogonida (Pantopoda). Structure and Evolution of Invertebrate Nervous Systems.

[49] Bergström J, Stürmer W, Winter G (1980). *Palaeoisopus*, *Palaeopantopus* and *Palaeothea*, pycnogonid arthropods from the Lower Devonian Hunsrück Slate, West Germany. Paläontol Z.

[50] Siveter DJ, Sutton MD, Briggs DEG, Siveter DJ (2004). A Silurian sea spider. Nature.

[51] Poschmann M, Dunlop J (2006). A new sea spider (Arthropoda: Pycnogonida) with a flagelliform telson from the Lower Devonian Hunsrück Slate, Germany. Palaeontology.

[52] Burris ZP (2011). The polygamous mating system of the sea spider *Achelia simplissima*. Invertebr Reprod Dev.

[53] Helfer H, Schlottke E (1935). Pantopoda. Dr. H. G. Bronns Klassen und Ordnungen des Tierreichs, Bd. 5, Abt. IV, Buch 2.

[54] Berry MB. The embryological development of *Achelia sawayai* (Ammotheidae, Pycnogonida), with notes on some phases of the behavior and anatomy of the adult, and on the phylogenetic position of the Pycnogonida. PhD thesis, Duke University; 1980.

[55] Scholtz G, Wolff C, Minelli A, Boxshall G, Fusco G (2013). Arthropod Embryology: Cleavage and Germ Band Development. Arthropod Biology and Evolution Molecules, Development, Morphology.

[56] Hartenstein V, Stollewerk A (2015). The evolution of early neurogenesis. Dev Cell.

[57] Jager M, Murienne J, Clabaut C, Deutsch J, Le Guyader H, Manuel M (2006). Homology of arthropod anterior appendages revealed by Hox gene expression in a sea spider. Nature.

[58] Manuel M, Jager M, Murienne J, Clabaut C, Le Guyader H (2006). Hox genes in sea spiders (Pycnogonida) and the homology of arthropod head segments. Dev Genes Evol.

[59] Minelli A, Fusco G, Minelli A, Boxshall G, Fusco G (2013). Arthropod Post-embryonic Development. Arthropod Biology and Evolution Molecules, Development, Morphology.

[60] Hoek PPC (1881). Nouvelles études sur les pycnogonides. Archives de Zoologie Expérimentale et Générale.

[61] Bogomolova EV (2007). Larvae of three sea spider species of the genus *Nymphon* (Arthropoda: Pycnogonida) from the White Sea. Russ J Mar Biol.

[62] Burris ZP (2011). Larval morphologies and potential developmental modes of eight sea spider species (Arthropoda: Pycnogonida) from the southern Oregon coast. J Mar Biol Assoc U K.

[63] Lehmann T, Weinzierl C, Melzer RR (2011). SEM description of the first larval instar of *Achelia assimilis* (Pycnogonida: Ammotheidae). J Mar Biol Assoc U K.

[64] Brenneis G, Ungerer P, Scholtz G (2008). The chelifores of sea spiders (Arthropoda, Pycnogonida) are the appendages of the deutocerebral segment. Evol Dev.

[65] Bogomolova EV, Malakhov VV (2003). Larvae of sea spiders (Arthropoda, Pycnogonida) from the White Sea. Entomol Rev.

[66] Miyazaki K, Suzuki H (1997). External morphology of the protonymphon larvae in a pycnogonid, *Ammothella biunguiculata* (Pycnogonida: Ammotheidae). Proc Arthropodan Embryological Soc Japan.

[67] Dogiel V (1911). Studien über die Entwicklungsgeschichte der Pantopoden. Nervensystem und Drüsen der Pantopodenlarven. Z Wiss Zool.

[68] Bain BA (2003). Postembryonic development in the pycnogonid *Austropallene cornigera* (Family Callipallenidae). Invertebr Reprod Dev.

[69] Dearborn GK. Post-embryonic development of the sea spider *Achelia gracilip*es (Chelicerata: Pycnogonida). Master thesis, Department of Biological Sciences, University of Alberta; 2003.

[70] Okuda S (1940). Metamorphosis of a pycnogonid parasistic in a hydromedusa. J Fac Sci Hokkaido Imperial Uni 6.

[71] Brenneis G, Scholtz G (2014). The ‘ventral organs’ of Pycnogonida (Arthropoda) are neurogenic niches of late embryonic and post-embryonic nervous system development. PLoS ONE.

[72] Miyazaki, Makioka T (2012). Postembryonic development of the female reproductive system in the pycnogonid *Propallene longiceps* (Pycnogonida, Callipallenidae). Invertebr Reprod Dev.

[73] Gillespie JM, Bain BA (2006). Postembryonic development of *Tanystylum bealensis* (Pycnogonida, Ammotheidae) from Barkley Sound, British Columbia, Canada. J Morphol.

[74] Dogiel V (1951). *Klass Mnogokolenchatykh (Pantopoda)* (Class of Sea Spiders (Pantopoda)). Treatise on Zoology.

[75] Bogomolova EV, Malakhov VV (2004). Fine morphology of larvae of sea spiders (Arthropoda: Pycnogonida) from the White Sea. Zoologiya Bespozvonochnykh.

[76] Cano E, López-González PJ (2009). Novel mode of postembryonic development in *Ammothea* genus (Pycnogonida: Ammotheidae) from Antarctic waters. Sci Mar.

[77] Fornshell JA, Ferrari FD (2012). Larvae of the pycnogonids *Ammothea gigantea* Gordon, 1932 and *Achelia cuneatis* Child, 1999 described from archived specimens. Arthropods.

[78] Fornshell JA (2014). Larvae of the pycnogonids *Ammothea striata* (Möbius, 1902) and *Ammothea carolinensis* Leach, 1814 described from archived specimens. Invertebr Biol.

[79] Ohshima H (1937). The Life-History of “*Nymphonella tapetis*” Ohshima (“Pantopoda, Eurycydidae”). Extrait des Comptes Rendus du XII Congress International de Zoologie - Lisbonne.

[80] Ogawa K, Matsuzaki K (1985). Discovery of bivalve-infesting Pycnogonida, *Nymphonella tapetis*, in a new host, *Hiatella orientalis*. Zool Sci.

[81] Salazar-Vallejo S, Stock JH (1987). Apparent parasitism of *Sabella melanostigma* (Polychaeta) by *Ammothella spinifera* (Pycnogonida) from the Gulf of California. Rev Biol Trop.

[82] Ohshima H (1933). Young pycnogonids found parasitic on nudibranchs. Annotationes Zoologicae Japonenses.

[83] Malakhov VV, Bogomolova EV (2001). The first finding of a sea spider (Pantopoda) planktonic larva. Dokl Biol Sci.

[84] Lebour MV (1916). Notes on the life history of *Anaphia petiolata* (Kröyer). J Mar Biol Assoc U K.

[85] Lebour MV (1945). Notes on the Pycnogonida of Plymouth. J Mar Biol Assoc U K.

[86] Maxmen A. Pycnogonid development and the evolution of the arthropod body plan. PhD thesis, Harvard University, Cambridge (Massachusetts); 2006.

[87] Lovely EC (2005). The life history of *Phoxichilidium tubulariae* (Pycnogonida: Phoxichilidiidae). Northeast Nat.

[88] Adlerz G (1888). Bidrag till pantopodernas morfologi och utvecklingshistoria. Bihang till Kungliga Svenska Vetenskaps-Akademiens Handlingar.

[89] Hilton WA (1916). The life history of *Anoplodactylus erectus* Cole. J Entomol Zool Pomona Coll Claremont.

[90] Russell D, Hedgpeth JW (1990). Host utilization during ontogeny by two pycnogonid species (*Tanystylum duospinum* and *Ammothea hilgendorfi*) parasitic on the hydroid *Eucopella everta* (Coelenterata: Campanulariidae). Bijdragen tot de Dierkunde.

[91] Lou T-H (1936). Notes sur *Lecythorhynchus hilgendorfi* Böhm (Pycnogonida). Contrib Inst Zool Natl Acadamy Peiping.

[92] Hooper J (1980). Some aspects of the reproductive biology of *Parapallene avida* Stock (Pycnogonida: Callipallenidae) from Northern New South Wales. Aust Zool.

[93] Hoek PPC (1881). Report on the Pycnogonida, dredged by H.M.S. Challenger during the years 1873–76. Challenger Rep Zool.

[94] Bamber RN (2007). A holistic re-interpretation of the phylogeny of the Pycnogonida Latreille, 1810 (Arthropoda). Zootaxa.

[95] Waloszek D, Dunlop JA (2002). A larval sea spider (Arthropoda: Pycnogonida) from the Upper Cambrian “Orsten” of Sweden, and the phylogenetic position of pycnogonids. Palaeontology.

[96] Arango CP, Wheeler WC (2007). Phylogeny of the sea spiders (Arthropoda, Pycnogonida) based on direct optimization of six loci and morphology. Cladistics.

[97] Arabi J, Cruaud C, Couloux A, Hassanin A (2010). Studying sources of incongruence in arthropod molecular phylogenies: Sea spiders (Pycnogonida) as a case study. Comptes Rendus Biologies.

[98] Dietz L, Mayer C, Arango CP, Leese F (2011). The mitochondrial genome of *Colossendeis megalonyx* supports a basal position of Colossendeidae with the Pycnogonida. Mol Phylogenet Evol.

[99] Chimenz Gusso C, Gravina MF (2001). Faunistic and biological traits of some Antarctic Pycnogonida. Italian Journal of Zoology.

[100] Nakamura K, Kano Y, Suzuki N, Namatame T, Kosaku A (2007). 18S rRNA phylogeny of sea spiders with emphasis on the position of Rhynchothoracidae. Mar Biol.

[101] Arango CP (2002). Morphological phylogenetics of the sea spiders (Arthropoda: Pycnogonida). Org Divers Evol.

[102] Stock JH (1978). Abyssal Pycnogonida from the north-eastern Atlantic Basin, Part 1. Cah Biol Mar.

[103] Child AC (1979). Shallow water Pycnogonida of the Isthmus of Panama and the coasts of Middle America. Smithson Contrib Zool.

[104] Munilla T. Evoluciøn y filogenia de los picnogønidos. In: Melic A, de Haro JJ, Mendez M, Ribera I, editors. Evoluciøn y filogenia de Arthropoda. Zaragoza; 1999. p. 273–279.

[105] Brenneis G. On the embryonic and post-embryonic development of *Pseudopallene* sp. (Arthropoda, Pycnogonida) with special focus on neurogenesis and nervous system differentiation. Doctoral thesis, Humboldt-Universität zu Berlin; 2013.

[106] Ferrari FD, Fornshell J, Vagelli AA, Ivanenko VN, Dahms H-U (2011). Early post-embryonic development of marine chelicerates and crustaceans with a nauplius. Crustaceana.

[107] Waloszek D, Maas A (2005). The evolutionary history of crustacean segmentation: a fossil-based perspective. Evol Dev.

[108] Liu Y, Melzer RR, Haug JT, Haug C, Briggs DEG, Hörnig MK, He Y, Hou X (2016). Three-dimensionally preserved minute larva of a great-appendage arthropod from the early Cambrian Chengjiang biota. Proc Natl Acad Sci U S A.

[109] Gnanamuthu CP (1950). Notes on the morphology and development of a pycnogonid, *Propallene kempi* (Calman), from Madras plankton. Proc Zoolog Soc Bengal.

[110] Wohlgemuth SD. The reproductive ecology and larval development of two species of pycnogonids, *Anoplodactylus lentus* Wilson and *Tanystylum orbiculare* Wilson (Pycnogonida, Pantopoda) from North Inlet, South Carolina. Master thesis, University of South Carolina, Department of Biology, 1979.

[111] Hedgpeth JW (1963). Pycnogonida of the North American Arctic. J Fish Res Board Can.

[112] Just J (1972). Revision of the genus *Boreonymphon* G. O. Sars (Pycnogonida) with a description of two new species, *B. ossiansarsi* Knaben and *B. compactum* Just. Sarsia.

[113] Arnaud F (1978). A new species of *Ascorhynchus* (Pycnogonida) found parasitic on an opisthobranchiate mollusc. Zool J Linnean Soc.

[114] Miyazaki K (2002). Occurrence of juvenile forms of a pycnogonid, *Ammothella biunguiculata* (Pycnogonida, Ammotheidae) in an Actinian, *Entacmaea actinostoloides* (Anthozoa, Stichodactylidae). Proc Arthropodan Embryological Soc Japan.

